# Zika Virus Induced More Severe Inflammatory Response Than Dengue Virus in Chicken Embryonic Livers

**DOI:** 10.3389/fmicb.2019.01127

**Published:** 2019-05-22

**Authors:** Zongyi Zhang, Menghan Sun, Jieping Deng, Jianhai Yu, Xuesong Yang, Wei Zhao, Guobing Chen, Pengcheng Wang

**Affiliations:** ^1^Department of Microbiology and Immunology, School of Basic Medical Sciences, Jinan University, Guangzhou, China; ^2^Guangdong Provincial Key Laboratory of Tropical Disease Research, School of Public Health, Southern Medical University, Guangzhou, China; ^3^Division of Histology and Embryology, Key Laboratory for Regenerative Medicine of the Ministry of Education, School of Basic Medical Sciences, Jinan University, Guangzhou, China

**Keywords:** Zika virus, Dengue virus, chicken embryo, liver, inflammation

## Abstract

Dengue (DENV) and Zika virus (ZIKV) are important *flaviviruses* in tropical and subtropical regions, causing severe Dengue Hemorrhagic Fever (DHF)/Dengue Shock Syndrome (DSS) and microcephaly, respectively. The infection of both viruses during pregnancy were reported with adverse fetal outcomes. To investigate the effects of ZIKV and DENV infections on fetal development, we established an infection model in chicken embryos. Compared with DENV-2, the infection of ZIKV significantly retarded the development of chicken embryos. High viral loads of both DENV-2 and ZIKV was detected in brain, eye and heart 7 and 11 days post-infection, respectively. Interestingly, only ZIKV but not DENV-2 was detected in the liver. Even both of them induced apparent liver inflammation, ZIKV infection showed a more severe inflammatory response than DENV-2 infection based on the inflammation scores and the gene expression levels of IL-1β, TNF, IL-6, and TGFβ-2 in liver. Our results demonstrated that ZIKV induced more severe inflammatory response in chicken embryo liver compared to DENV-2, which might partially attribute to viral replication in liver cells. Clinicians should be aware of the potential liver injury associated with ZIKV infection in patients, especially in perinatal fetuses.

## Introduction

Dengue virus (DENV) is one of the most important arboviruses in tropical and subtropical regions, and it belongs to the family *Flaviviridae*, including four distinct serotypes designated DENV-1, DENV-2, DENV-3, and DENV-4, which cause mild Dengue Fever and more severe Dengue Hemorrhagic Fever (DHF) and Dengue Shock Syndrome (DSS) (Halstead, 1989). Besides the febrile disease in general population caused by DENV, there have been concerns about the effects on fetuses due to the viral infection in pregnant women. Many case reports and cohort studies suggest the association of DENV infection during pregnancy with adverse fetal outcomes, including prematurity and low birthweight (Paixao et al., 2016; Sharma et al., 2016), while the exact linkage and mechanisms are still under debate. First discovered in 1947 in Uganda, Zika virus (ZIKV) only caused sporadic infections in Africa and Southeast Asia, until the major outbreaks in Micronesia in 2007 and Polynesia in 2013, which started to draw significant attentions (Faye et al., 2014; Sampathkumar and Sanchez, 2016). In 2015, another widespread outbreak in Brazil made ZIKV a major international public health concern (Lessler et al., 2016). As a member of *flavivirus*, ZIKV is mainly transmitted through the bites of infected mosquitos, and the consequences of the infection range from asymptomatic to non-specific symptoms in adults, including fever, rash and headaches. However, the infection in pregnant women could cause severe neurological damages, like microcephaly, in fetuses (Driggers et al., 2016; Mlakar et al., 2016). It’s reported that ZIKV could infect placental trophoblasts (Tabata et al., 2016; Aagaard et al., 2017; Sheridan et al., 2017) and virus genome was found in the tissues of infected mothers and neonates with microcephaly (Calvet et al., 2016; Melo et al., 2016), which indicated that ZIKV could be vertically transmitted, probably in a transplacental manner, but the mechanisms of how exactly the virus is passed to the fetuses are still obscure.

Liver involvement is commonly seen in DENV infection, with hepatocytes and Kupffer cells as the primary targets (Seneviratne et al., 2006). The histological changes during DENV infection include hepatocellular necrosis, Kupffer cell hyperplasia and destruction, and mononuclear cells infiltration. It is reported that severe liver injury is seen in DHF and DSS cases (Huerre et al., 2001; Tan and Bujang, 2013; Yudhishdran et al., 2014; Dalugama and Gawarammana, 2017). A cytokine storm could be induced during DENV infection with the remarkably increased cytokines, such as IL-6 and TNF (Chaturvedi et al., 1999), which is believed to be responsible for severe DHF and DSS. However, the exact mechanisms of the liver injury are still unclear. Even the infection of ZIKV has been reported in multiple organs and systems, including hemolymphatic tissues, cardiopulmonary, gastrointestinal, and genitourinary tissues (Coffey et al., 2017), there are very few reports about the liver damage in ZIKV infection so far. Recently, severe liver injury in ZIKV infected adult was reported (Wu et al., 2017), and a postmortem study showed lymphocytic infiltration in livers of congenitally infected neonates, indicating the liver involvement in fetuses (Sousa et al., 2017). However, how the virus causes the liver injury, and its role in fetal development still needs more investigation.

In this study, chicken embryos were inoculated with DENV-2 and ZIKV, respectively. The effects of viral infection on fetal development and liver involvement were evaluated. The experimental evidences were provided for investigating the mechanisms of the adverse effects DENV and ZIKV on fetuses and the induced liver injury.

## Materials and Methods

### Viruses

Dengue virus serotype 2 New Guinea C strain (DENV-2 NGC, GenBank #: NC_001474), and Zika virus Z16006 strain (ZIKV, GenBank #: KU955589.1) were used for chicken embryo infection. Zika virus Z16006 strain was obtained from the Institute of Microbiology in the Center for Disease Control and Prevention of Guangdong Province, China. It was isolated in February 2016 in China from a patient who traveled to Fiji and Samoa, where ZIKV epidemics was then ongoing (Wu et al., 2016). C6/36 cells were cultured in RPMI-1640 medium (10% FBS, 1% PS) at 28°C and inoculated with DENV or ZIKV, respectively. When CPE reached 70%, the cell culture was frozen and thawed for three times, and the supernatant was collected with centrifugation at 10,000 rpm for 5 min. The virus titer was determined with plaque forming assay in Vero cells. The manipulation of viruses and virus contaminated materials were performed in standard biosecurity procedures in our BSL-3 laboratory certified by Health Commission of Guangdong Province, China.

### Chick Embryos and Manipulation

Fertilized chicken eggs were purchased from the Avian Farm of the South China Agricultural University.

The handling of embryonic eggs complied with the “Guidelines on Ethical Treatment of Experimental Animals” (2006) No. 398 set by the Chinese Ministry of Science and Technology, and the protocol was approved by the Committee on the Ethics of Animal Experiments of Jinan University. The eggs were incubated in a humidified incubator (Yiheng Instrument, Shanghai, China) at 37°C and 50–60% humidity until the required Hamburger-Hamilton stage (Hamburger and Hamilton, 1951). The virus inoculation was performed following a protocol described before with some modifications (Goodfellow et al., 2016). Briefly, three milliliters of albumen was removed on embryonic day 2 (E2) and the embryos were kept in the incubator for 3 more days. The eggshell was sterilized with ethanol and windowed to visualize the embryo, and the virus was injected with a Hamilton Microliter syringe on E5. Two μl of normal saline containing DENV-2 (0, 200, and 10^4^ PFU) or ZIKV (5, 50, and 500 PFU) was injected into the brain vesicle. Eggs were sealed with sterile tape and immediately returned to the incubator, observed every day and the embryos were harvested at the desired time points.

### Real-Time PCR Assay

Post-viral inoculation, various organs, including brains, eyes, hearts, and livers, were harvested at different time points. Organ samples were washed with PBS three times, put in 1.5 ml Eppendorf tubes and weighed. The samples were homogenized with stainless steel beads, and the total RNA was extracted using the E.Z.N.A^®^ Total RNA Kit (OMEGA, Georgia, United States). The viral RNA was quantified with real-time PCR by using the PrimeScript^TM^ RT Reagent Kit (Takara, Japan). The reverse transcription reactions were performed in Bio-Rad S1000^TM^ thermocycler (Bio-Rad, United States). For viral RNA amplification, qPCR mixture was prepared in 20 μl volume, containing 10 μl Bestar qPCR Master Mix, PCR forward and reverse primers (10 uM with 0.4 μl each), 0.2 μl PCR probe (10 uM) and 1 μl DNA template. The mixture was then incubated at 95°C for 2 min, followed by 40 PCR cycles (95°C for 10 s, 60°C for 30 s), using ABI 7000 Real-Time PCR machines. The viral loads in tissues were calculated by using standard curves obtained from serial dilutions of DENV-2 or ZIKV stocks titrated with plaque assays in Vero cells (Yu et al., 2017) and expressed as plaque-forming units per milliliter equivalents (PFUeq/ml) (Thomas et al., 2010; de la Cruz-Hernandez et al., 2013). The expression levels of various inflammation cytokines, including IL-1β, IL-6, TGFβ-2, and TNF were also determined with qPCR. qPCR mixture was prepared in 20 μl volume, containing 10 μl SYBR Premix EX TaqII (Tli RNaseH Plus), PCR forward and reverse primers (10 uM with 0.4 μl each), and 1 μl DNA template. The mixture was then incubated at 95°C for 3 min, followed by 40 PCR cycles (95°C for 5 s, 60°C for 20 s, 72°C for 20 s). Corresponding relative mRNA expression was calculated by the 2^-ΔΔCq^ method and normalized to GAPDH. The qPCR results are representative of three independent experiments and the chicken specific primers used were described previously (Meriwether et al., 2010; Zhi-qin et al., 2014; Long et al., 2018) and showed in Table 1.

**Table 1:** Primer sequences for qPCR.

Primer name	Primer sequence (5′-3′)	Length	Location	Genbank accession numbers
IL-1β-F	ATGTCGTGTGTGATGAGCG	19	579–587	NM_204524
IL-1β-R	CTTGTAGGTGGCGATGTTGA	20	821–802	
IL-6-F	GATGTGCAAGAAGTTCACCG	20	286–305	HM179640
IL-6-R	TGGCAGATTGGTAACAGAGG	20	787–768	
TGFβ-2-F	CTCTGGGCAGGGAGATGTATG	20	1835–1855	NM_205428
TGFβ-2-R	CAATCTCATTCCTGAGAAGTGCTA	20	1956–1937	
TNF-F	CCGTAGTGCTGTTCTATGACCG	22	362–383	AY765397
TNF-R	GTTCCACATCTTTCAGAGCATCAA	24	550–527	
GAPDH-F	GGTGGTGCTAAGCGTGTTA	19	391–409	NM_204305
GAPDH-R	CCCTCCACAATGCCAA	16	569–554	
DENV-2 Probe	AAGTAACACCACAGAGTTCCATCACA	26	1424–1168	KM204118.1
DENV-2 -F	CAGTCGGAAATGACACAG	18	1385–1402	
DENV-2 -R	GCAACACCATCTCATTGA	18	1531–1514	
ZIKV Probe	FAM-AGGTGAAGCCTACCTTGACAAGC ARTCA-BHQ	28	1141–1168	KU955589.1
ZIKV-F	CVGACATGGCTTCGGACAGY	20	1107–1126	
ZIKV-R	CCCARCCTCTGTCCACYAAYG	21	1214–1194	


### Histochemical Analysis

The fresh livers were harvested and then fixed in 4% paraformaldehyde, dehydrated, embedded in paraffin wax and serially sectioned at 5 μm. Haematoxylin and eosin (H&E) staining was performed to observe histological changes and inflammation. The inflammation severity was evaluated based on the percentage of the perivascular inflammatory cell count relative to the total cells in the same area (Duan et al., 2004). Briefly, the score system was: 0, no inflammatory cells; 1, inflammatory cell count <25%; 2, inflammatory cell count between 25 and 50%; 3, inflammatory cell count between 50 and 75%; 4, inflammatory cell count >75%. Blind examination was performed and a minimum of three randomly selected images from at least four embryo liver samples were assessed per group per assigned time point.

### Data Analyses

Data analyses were performed using Graphpad Prism 5 and SPSS 13.0 software package. The results are presented as Mean ± SD. Statistical analyses were performed using one-way ANOVA and Student’s *t*-test. *P* < 0.05 was considered to be statistically significant.

## Results

### DENV-2 and ZIKV Infection Suppressed Chicken Embryo Development

**FIGURE 1:**
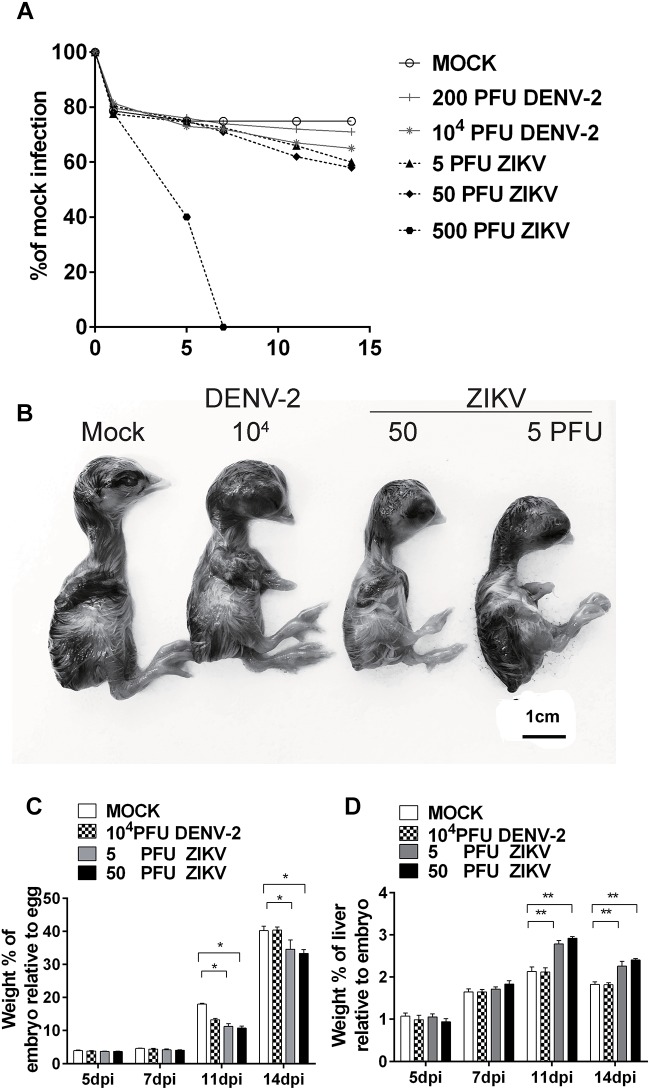
DENV-2 and ZIKV infection significantly retarded chicken embryo development. **(A)** Survival rates of chicken embryos upon viral infection. DENV-2 and ZIKV were injected into the brain vesicle of chicken embryos on E5, respectively, and embryo viability was checked daily. **(B)** Morphological appearance of embryonic chicks after viral infection. At 11 dpi, chicks were harvested to check morphological changes and the viral infection induced smaller sized embryos. **(C)** Weight percentages of chicken embryo relative to whole egg after viral infection. Eggs and embryos were weighed to calculate the weight percentage of embryos relative to eggs at desired time points. Viral infection significantly reduced the weight percentage of chicken embryos relative to eggs at 11 and 14 dpi. **(D)** Weight percentages of embryo liver relative to whole embryo after viral infection. Livers were collected and weighed to calculate the weight percentage of livers relative to whole embryos at desired time points. ZIKV infection significantly increased the weight percentage of livers relative to embryos at 7, 11, and 14 dpi. Each experiment was repeated at least three times. ^∗^*P* < 0.05. ^∗∗^*P* < 0.01. Scale bar = 1 cm.

Chicken embryos were infected with DENV-2 NGC strain or a ZIKV isolated from a human subject traveled to the epidemic area. With a windowing procedure to access to the embryos, different doses of viruses were injected into the brain vesicle on E5. Daily monitoring of embryo viability revealed no death due to the viral inoculation, except for the high dose 500 PFU ZIKV group, in which all embryos died at 7 days post-infection (dpi) (Figure 1A). We then assessed the morphology of embryos and found that the embryo development was significantly altered upon viral inoculation. In the 10^4^ PFU DENV-2 group, chicken embryos were smaller than those in mock (Figure 1B), and embryo weight was decreased as well at 11 dpi (Figure 1C). In both 5 and 50 PFU ZIKV groups, the chicken embryos were significantly smaller than those in mock at 11 and 14 dpi (Figure 1B), and embryo weight was significantly decreased at the same time (Figure 1C). Furthermore, ZIKV infection significantly increased liver weight, indicating an ongoing inflammatory reaction in the liver (Figure 1D). Since brains are the major target for ZIKV infection in fetuses, the chicken brain size was also measured, and the result showed that ZIKV induced an obviously reduced brain size in 50 PFU group (Supplementary Figure S1), which was consistent with the previous report in chicken embryos (Goodfellow et al., 2016). In our Department, another research group performed immuno-fluorescent analysis of pHIS3 and cleaved caspase-3, and demonstrated that ZIKV infection mediated suppression of cell proliferation and increase in apoptosis and inhibited the cranial neural crest production in chicken embryos (Yan et al., 2019). All these indicated the successful establishment of our model and the viral infection did suppress chicken embryo development.

### DENV-2 and ZIKV Replicated in Multiple Organs in Chicken Embryos

**FIGURE 2:**
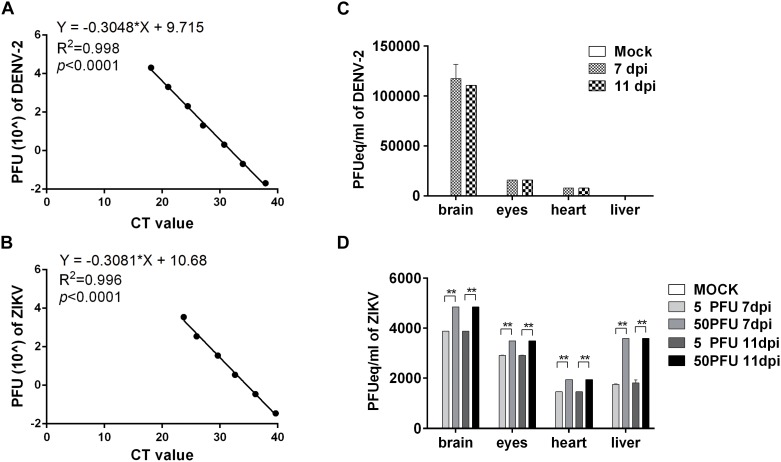
DENV-2 and ZIKV replicated in chicken embryos. **(A,B)** q-PCR standard curve for DENV-2 **(A)** and ZIKV **(B)**. By using a series of diluted DENV-2 **(A)** or ZIKV **(B)** stock as standards, a formula was deduced for tissue viral load calculation. **(C)** The viral load of DENV-2 in various organs in chicken embryos. The chicken embryos were inoculated with 10^4^ PFU DENV-2 and samples were collected at 7 and 11 dpi, respectively. Viral RNA was extracted and quantified with qPCR. The tissue viral load was calculated with the deduced formula and expressed as PFUeq/ml. **(D)** The viral load of ZIKV in various organs in chicken embryos. The chicken embryos were inoculated with five PFU and 50 PFU ZIKV, and samples were collected at 7 and 11 dpi, respectively. Viral RNA was extracted and quantified with qPCR. The tissue viral loads were calculated with the deduced formula and expressed as PFUeq/ml. *n* = 3, ^∗^*P* < 0.05. ^∗∗^*P* < 0.01.

To determine whether the viruses replicated in the embryos, the brains were harvested at 7 and 11 dpi, respectively, to quantify the viral RNA using qRT-PCR with a series of dilution of virus stock suspension used as standards. The q-PCR curves for diluted DENV-2 and ZIKV standards are shown in Figure 2A,B, respectively, and the viral copies in tissues were expressed as PFU per milliliter equivalents (PFUeq/ml). The results showed that, compared with mock group, the calculated viral copy numbers in 10^4^ PFU DENV-2 group, 5 PFU ZIKV group and 50 PFU ZIKV group increased at least 20-folds compared to the initial inoculums, indicating the viral replication in the embryos (Figure 2C,D). Furthermore, other organs from these embryos were collected for virus detection. In the 10^4^ PFU DENV-2 group, the virus was detected in the eyes and the hearts, but not in the livers in embryos at 7 and 11 dpi. For ZIKV virus, however, there were remarkable viral load in all tested organs for both groups (Figure 2C,D). Meanwhile, immunohistochemical assay and immunofluoresent staining revealed the expression of ZIKV E protein in both brain and liver cells (Supplementary Figures S2, S3).

### DENV-2 Inoculation Induced Inflammation in Chicken Embryo Livers

**FIGURE 3:**
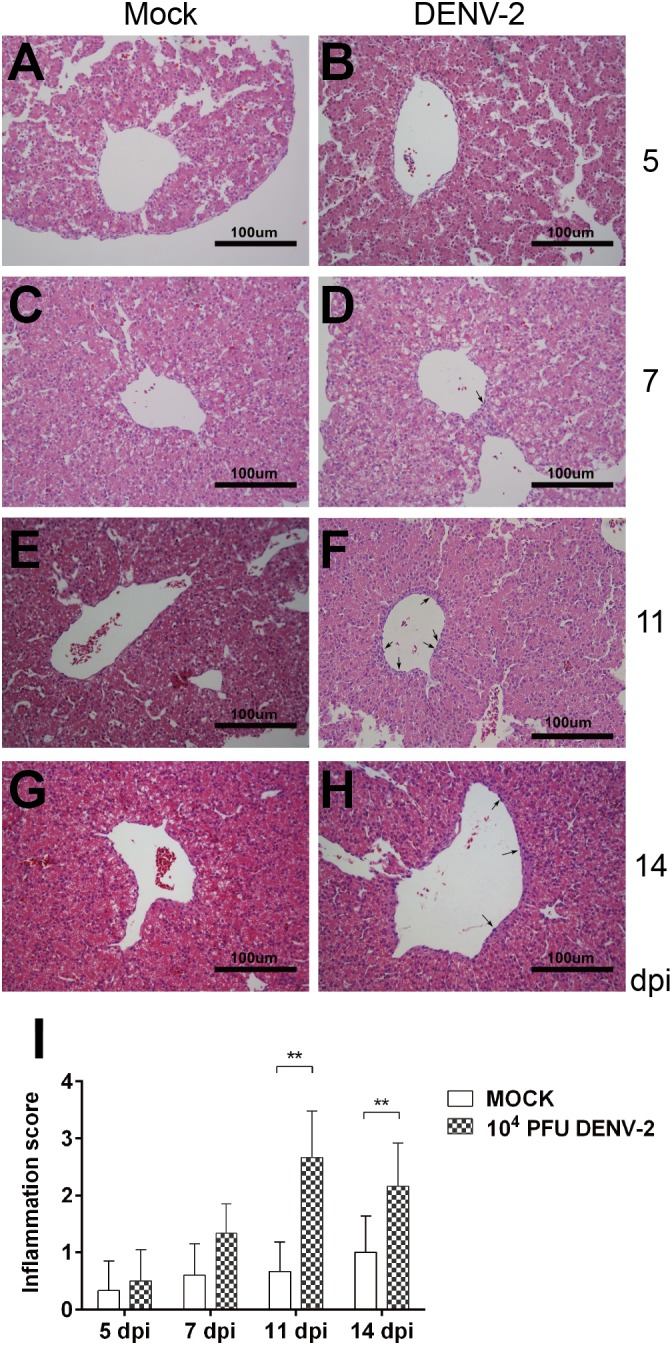
DENV-2 infection induced inflammatory response in chicken embryonic livers. Chicken embryos were inoculated with 10^4^ PFU DENV-2 and liver samples were collected at 5, 7, 11, and 14 dpi, respectively. Liver tissue was sectioned at 5 μm and HE staining was performed for histological analysis. **(A,C,E,G)** No inflammatory reaction was observed in livers in Mock at 5, 7, 11, and 14 dpi, respectively. **(B)** No inflammatory reaction was observed in DENV-2 infected chicken embryonic liver at 5 dpi. **(D)** A few inflammatory cells were observed in perivascular area in DENV-2 infected chicken embryonic liver at 7 dpi. **(F)** A large amount of inflammatory cells were observed in perivascular area in DENV-2 infected chicken embryonic liver at 11 dpi. **(H)** Some inflammatory cells were observed in perivascular area at 14 dpi, and the inflammation was subsided compared to that at 11 dpi. **(I)** Inflammatory scores of chicken embryonic livers at 5, 7, 11, and 14 dpi, respectively. *n* = 3, ^∗^*P* < 0.05. ^∗∗^*P* < 0.01.

Liver is the most common target organ involved in Dengue virus infection. The doses we used didn’t cause any embryo death, or apparent symptoms. We then carried out histological analysis of the liver sections. The H and E staining revealed no pathological changes in mock embryo livers during the whole course. In 10^4^ PFU DENV-2 inoculated embryos, the inflammatory response was not found at 5 dpi, but at 7, 11, and 14 dpi (Figure 3). There were a few inflammatory cells, mainly monocytes, in the perivascular area at 7 dpi. The monocyte infiltration was significantly higher than that in mock at both 11 and 14 dpi, while the inflammation reduced at 14 dpi compared to 11 dpi (Figure 3).

### ZIKV Inoculation Induced Inflammation in Chicken Embryo Livers

**FIGURE 4:**
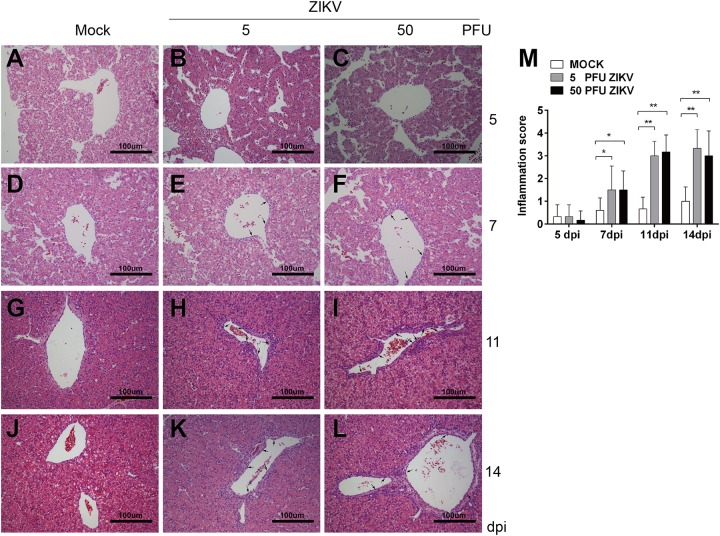
ZIKV infection induced inflammatory response in chicken embryonic livers. Chicken embryos were inoculated with 5 PFU or 50 PFU ZIKV, and liver samples were collected at 5, 7, 11, and 14 dpi, respectively. Liver tissue was sectioned at 5 μm and HE staining was performed for histological analysis. **(A,D,G,J)** No inflammatory reaction was observed in livers in Mock at 5, 7, 11, and 14 dpi, respectively. **(B,C)** No inflammatory reaction was observed in 5 PFU ZIKV or 50 PFU ZIKV infected chicken embryonic livers at 5 dpi. **(E)** A few inflammatory cells were observed in perivascular area in 5 PFU ZIKV infected chicken embryonic liver at 7 dpi. **(F)** A few inflammatory cells were observed in perivascular area in 50 PFU ZIKV infected chicken embryonic liver at 7 dpi. **(H,I)** A large amount of inflammatory cells were observed in perivascular area in 5 PFU ZIKV and 50 PFU ZIKV infected chicken embryonic liver at 11 dpi, respectively. **(K,L)** A large amount of inflammatory cells were observed in perivascular area in 5 PFU ZIKV and 50 PFU ZIKV infected chicken embryonic liver at 14 dpi, respectively. **(M)** Inflammatory scores of chicken embryonic livers at 5, 7, 11, and 14 dpi, respectively. *n* = 3, ^∗^*P* < 0.05. ^∗∗^*P* < 0.01.

Some clinical case reports showed that the infection of ZIKV induced hepatic injury similar to DENV. In our study, the inoculation of 500 PFU ZIKV led to all embryos death. When lower virus doses (5 and 50 PFU) used, no virus induced death was observed. At 11 and 14 dpi, liver weights in both ZIKV groups were dramatically increased compared to mock (Figure 1D). Similar to that in DENV-2 group, there was no inflammatory response at 5 dpi and monocyte infiltration was observed at 7, 11, and 14 dpi in both ZIKV groups (Figure 4). A few inflammatory cells gathered in perivascular area at 7 dpi and it’s significantly increased at 11 dpi. Unlike that in DENV-2 group, the cell counts remained at high level, indicating a more severe and prolonged inflammation induced by ZIKV infection.

### Profiling of Inflammation Related Cytokines in Chicken Embryonic Livers

**FIGURE 5:**
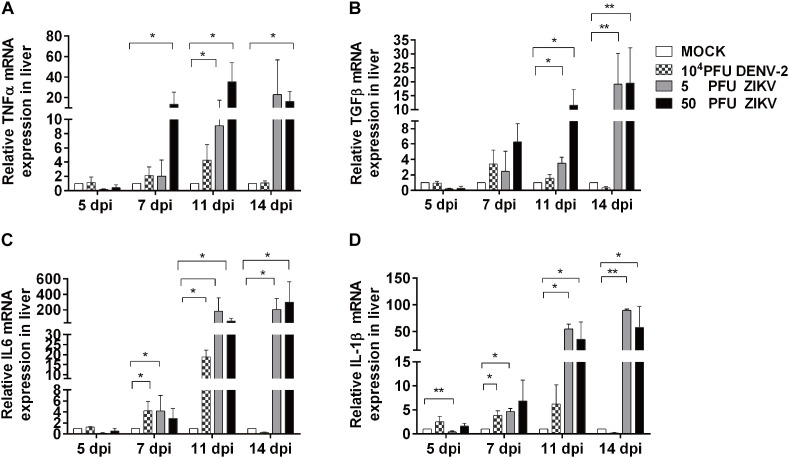
DENV-2 and ZIKV infection induced up-regulation of inflammatory cytokine expression in chicken embryonic livers. Total RNA was extracted from liver tissue and qPCR was performed to determine mRNA expression levels of various inflammation related cytokines at 5, 7, 11, and 14 dpi, respectively. *n* = 3, ^∗^*P* < 0.05. ^∗∗^*P* < 0.01.

To further evaluate the inflammatory response in viral infected chicken embryo livers, the mRNA expression levels of inflammatory cytokines were determined by using qPCR. As shown in Figure 5, the expression levels of IL-1β, TNF, IL-6, and TGFβ-2 in all virus groups were similar to those in mock at 5 dpi, which was consistent to the histological observation in liver sections. In DENV-2 group, IL-1β and IL-6 levels were significantly increased at 7 dpi, reached the highest level at 11 dpi, and returned to the same levels as those in mock at 14 dpi. Meanwhile, TNF and TGFβ-2 levels showed an increased trend and gradually returned to normal at 14 dpi. For two ZIKV groups, all tested cytokines showed significant increase from 7 dpi compared to mock, and continuously rise and maintained at significantly high levels till 14 dpi, which confirmed a more severe and prolonged inflammatory response in the livers.

## Discussion

As one of the most important arboviruses, DENV causes febrile diseases in tropical and subtropical regions, resulting in symptoms like fever, headache, joint and retro-orbital pains, skin rash and occasionally more severe deep bleeding and shock syndrome. Multiple organ systems could be affected, with the commonest being liver, leading to manifestation from asymptomatic elevated transaminase levels to acute liver failure (ALF). ZIKV also belongs to *flavivirus* genus and draws great attention since 2015 because of its infection in pregnant women potentially leading to severe neurological mal-development, microcephaly, in fetuses. It has been reported that the infection of ZIKV in adults could cause febrile symptoms similar to Dengue and resultant liver dysfunction (Coffey et al., 2017; Wu et al., 2017). Many clinical reports indicate that the DENV infection in pregnant women is a relevant factor to adverse fetal outcomes, while the exact linkage is still unclear. The infection of ZIKV in placental cells suggested that the virus could be transmitted vertically from the mothers, while its mechanisms and the role in fetal mal-development are unknown. An early study reported that ZIKV could not infect chicken embryonic cells in culture, but recently, it’s shown that the inoculation of ZIKV in chicken embryos led to microcephaly and viral replication in multiple organs (Goodfellow et al., 2016), which suggested that chicken embryos could be used as an *in vivo* model to study the role of ZIKV and even other *flaviviruses* in the pathogenesis of multiple systems, including liver. In this study, we attempted to establish the infection model of DENV and ZIKV in chicken embryos, and further explored the virus induced pathogenic changes in the organs. After direct injection in the brain vesicle, both DENV-2 and ZIKV were detected in multiple organs. For DENV-2, the virus RNA was not detectable in any tested tissues with a 200 PFU inoculum. However, high copies of viral gene were detected in the brain when 10^4^ PFU viruses were inoculated in the embryos at 7 and 11 dpi, respectively. Meanwhile, viral gene was detected in eyes and hearts as well, although it could not completely rule out the possibility that the viral RNA seen in eyes and hearts might be due to input virus form brain. For ZIKV, the highest 500 PFU dose led all embryos to death at 7 dpi, and the lower 5 and 50 PFU doses didn’t affect the survival rate compared to the mock. Subsequently, we assessed the viral gene expression levels in various organs of embryos in 5 and 50 PFU groups. It was observed that, besides in the brain, high levels of viral gene expression were detected in the eyes, hearts and livers. Meanwhile, the infection of both viruses induced smaller sized embryos, indicating that direct viral infection in fetuses might be part of the mechanisms for the adverse effects on fetal development. Thus, the successful establishment of DENV-2 and ZIKV infection in chicken embryos provided us an *in vivo* model to further study the influence of viral infection on development and related pathogenesis in fetuses.

Liver is one of the targets in human DENV infection, and recently clinical case reports showed that ZIKV infection could also cause liver injury, while the mechanisms and the role of induced inflammatory factors are not clear (Wu et al., 2017). Although no DENV-2 viral replication was detected in the liver, our result revealed that the inflammation response was observed in livers from 10^4^ PFU DENV-2 infected embryos at 7 dpi, and the inflammation continued to rise and reached to higher levels at 11 dpi and reduced at 14 dpi. We further detected the gene expression levels of inflammatory cytokines in liver using qPCR. Compared to the mock, the expression levels of IL-1β, TNF, and IL-6 were significantly up-regulated in DENV-2 group at 7 dpi, reached to the highest levels at 11 dpi, and reduced to normal level at 14 dpi, which was in consistence with the inflammatory responses observed in the livers. Franca et al. (2010) inoculated BALB/c mice with DENV-2 and found a similar pattern in the liver injury. They found that the inflammatory cell infiltration and Kupffer cell proliferation in the mouse liver started to increase on 5 dpi and reached to highest level on 7 dpi. The inflammation was alleviated from 14 dpi and turned to normal by 21 dpi, which was consistent with the changes in expression levels of IL-1β and TNF (Franca et al., 2010). We performed the same experiments using a DENV-1 strain and got similar results (Supplementary Figure S4). It’s generally believed that there are about 10^4-5.4^ PFU DENV virions entering the host by mosquito bites in the skin naturally (Bente and Rico-Hesse, 2006). Franca et al. (2010) used a lower dose for inoculation and detected no viral replication in the livers at any stage of the experiment, while in other studies, the viral replication in liver was observed with higher inoculation doses used (Paes et al., 2009), and the presence of virus in hepatocytes and Kupffer cells was often accompanied with more severe liver injury, alike in fatal Dengue cases (Couvelard et al., 1999; Rosen et al., 1999). These reports suggested that the virus could be cleared promptly by host immunity when low dose inoculation was performed, and the virus didn’t infect the hepatocytes and the inflammation was caused by the inflammatory mediators due to the viral infection. When high titer of virus was present, the cytolytic effects direct from the virus infection and replication could contribute to the liver injury as well (Dissanayake and Seneviratne, 2018).

In livers of 5 and 50 PFU ZIKV infected embryos, the expression levels of IL-1β, TNF, TGFβ-2 and IL-6 were all up-regulated significantly at 7 dpi, and maintained at high levels at 11 and 14 dpi, which were 10–20 folds higher than those in DENV-2 group. This is also consistent with the changes in inflammatory cell infiltration in livers. Unlike in DENV-2 group, the inflammation induced by ZIKV maintained at high level even at 14 dpi. Meanwhile, high copies of ZIKV viral RNA were detected in livers at 7 and 11 dpi. All these data indicated that a more severe liver injury was induced by ZIKV than DENV-2.

Due to the devastating consequences of microcephaly, most of the studies on the effects of ZIKV in fetuses have been focused on the neural development, and there are very few reports about its effects on other systems. In this study, we demonstrated that the inoculation of DENV-2 and ZIKV in chicken embryos suppressed fetal development and induced inflammatory responses in livers. Although the amount of ZIKV used was much less, it caused more severe liver injury, which might be due to the effects of both viral replication and induced immune response. Our data provided experiment basis for understanding the mechanisms of the adverse effects caused by DENV and ZIKV on fetal development, and raised an alert to monitor the possible fetal liver injury in *flaviviruses*, especially ZIKV infected pregnant women.

## Ethics Statement

This study was carried out in accordance with the recommendations of “Guidelines on Ethical Treatment of Experimental Animals” (2006) No. 398 set by the Chinese Ministry of Science and Technology. The protocol was approved by the Committee on the Ethics of Animal Experiments of Jinan University.

## Author Contributions

ZZ performed most of the work. MS, JD, and JY participated in chicken embryo manipulation, virus amplification, and titration. GC and PW contributed to the conception of the experiment and the design. GC, PW, WZ, and XY drafted the manuscript for important intellectual content.

## Conflict of Interest Statement

The authors declare that the research was conducted in the absence of any commercial or financial relationships that could be construed as a potential conflict of interest.

## Supplementary Material

FIGURE S1 ZIKV inoculation induced smaller brain than DENV in chicken embryos. Chicken embryos were inoculated with virus or saline with brain injection at E5. Brains were harvested at 11 dpi to check morphological changes and the 50 PFU ZIKV infection induced obviously smaller brain size than other groups.

FIGURE S2 ZIKV was detected in chicken embryonic livers with immunohistochemistry. Chicken embryonic livers were harvested at 11 dpi, and immunohistochemical assay was performed to probe ZIKV Envelop protein. In brief, tissue sections at 5 μm were de-waxed in xylene, rehydrated and heated for antigen retrieval. After being immersed in 3% hydrogen peroxide for 25 min to block endogenous peroxidase, the sections were blocked with 3% BSA for 30 min at room temperature and incubated with primary antibodies against ZIKV Envelop protein (1:100, BF-1176-56, BioFront Technologies Inc.,) at 4°C overnight with shaking. The sections were then incubated with HRP labeled secondary antibodies (1:200; Invitrogen) at room temperature for 50 min in a dark box and finally visualized with DAB. All the sections were later counterstained with hematoxylin. A minimum of 3 randomly-selected images from 5 samples were assessed per group.

FIGURE S3 ZIKV was detected in chicken embryonic livers with immunefluoresent staining. Chicken embryonic livers were harvested at 11 dpi, and immunofluoresent staining was performed to probe ZIKV Envelop protein. In brief, tissue sections at 5 μm were de-waxed in xylene, rehydrated and heated for antigen retrieval. After being immersed in 3% hydrogen peroxide for 10 min to block endogenous peroxidase, the sections were blocked with 5% inactivated goat serum for 30 min at room temperature and incubated with primary antibodies against ZIKV Envelop protein (1:100, BF-1176-56, BioFront Technologies Inc.,) at 4°C overnight with shaking. The sections were then incubated with the corresponding Alexa Fluor CY3 labelled secondary antibodies (1:300; Invitrogen) at room temperature for 2 hours in a dark box. All the sections were later counterstained with DAPI (1:1000; Invitrogen) at room temperature for 30 min. A minimum of 3 randomly-selected images from 5 samples were assessed per group.

FIGURE S4 DENV-1 replicated in chicken embryos and induced inflammatory response in embryonic livers. Chicken embryos were inoculated with 1000 PFU DENV-1 virus (Hawaii strain, GenBank #: KM204119) with brain injection at E5, and various organs were harvested at 11 dpi for assays. **(A)** The viral load of DENV-1 in various organs in chicken embryos. Viral RNA was extracted and quantified with qPCR. The viral loads in tissues were calculated by using standard curves obtained from serial dilutions of DENV-1 stocks titrated with plaque assays in Vero cells and expressed as plaque-forming units per milliliter equivalents (PFUeq/ml). **(B)** Liver tissue was sectioned at 5 μm and HE staining was performed for histological analysis. A large amount of inflammatory cells were observed in perivascular area in DENV-1 infected chicken embryonic liver. **(C)** Total RNA was extracted from liver tissue and qPCR was performed to determine mRNA expression levels of various inflammation related cytokines. TNF- and IL-6 showed significant increase compared to mock.
