# Supplementary figures and images for: Zika Virus Induced More Severe Inflammatory Response Than Dengue Virus in Chicken Embryonic Livers
Source: Front Microbiol. 2019 May 22;10:1127. doi: 10.3389/fmicb.2019.01127 (PMC6540742; doi:10.3389/fmicb.2019.01127)

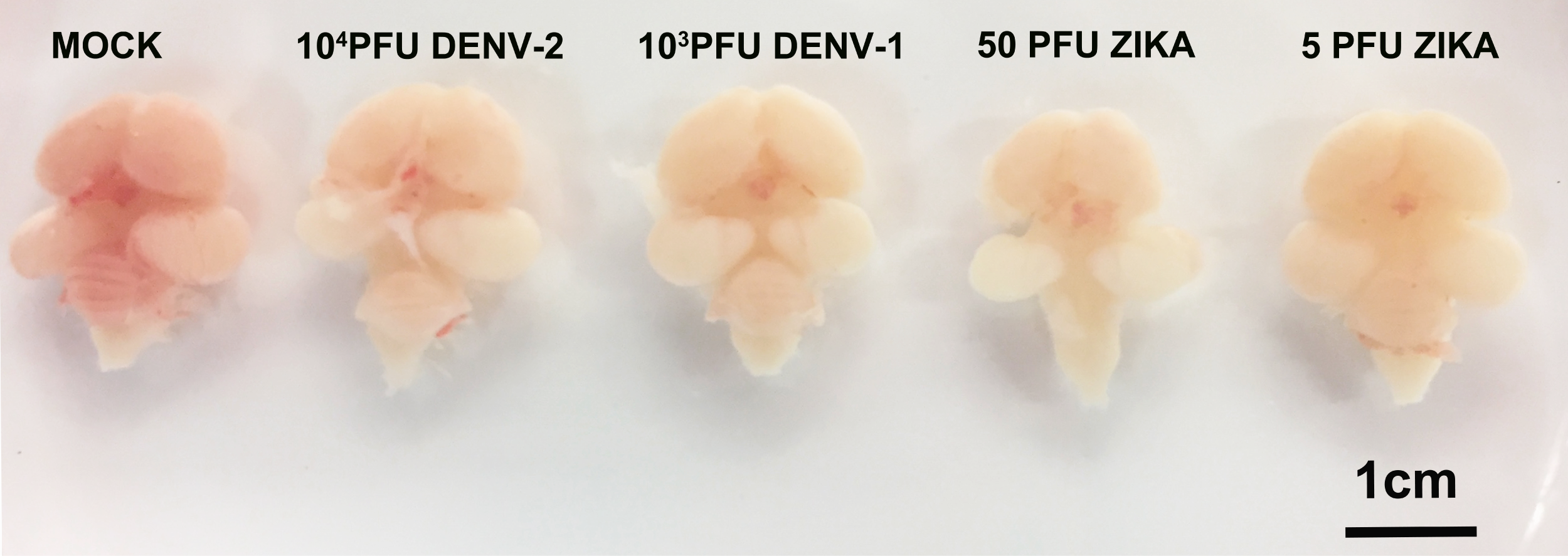

Supplement: FIGURE S1 — ZIKV inoculation induced smaller brain than DENV in chicken embryos. Chicken embryos were inoculated with virus or saline with brain injection at E5. Brains were harvested at 11 dpi to check morphological changes and the 50 PFU ZIKV infection induced obviously smaller brain size than other groups. [file Image_1.TIF]

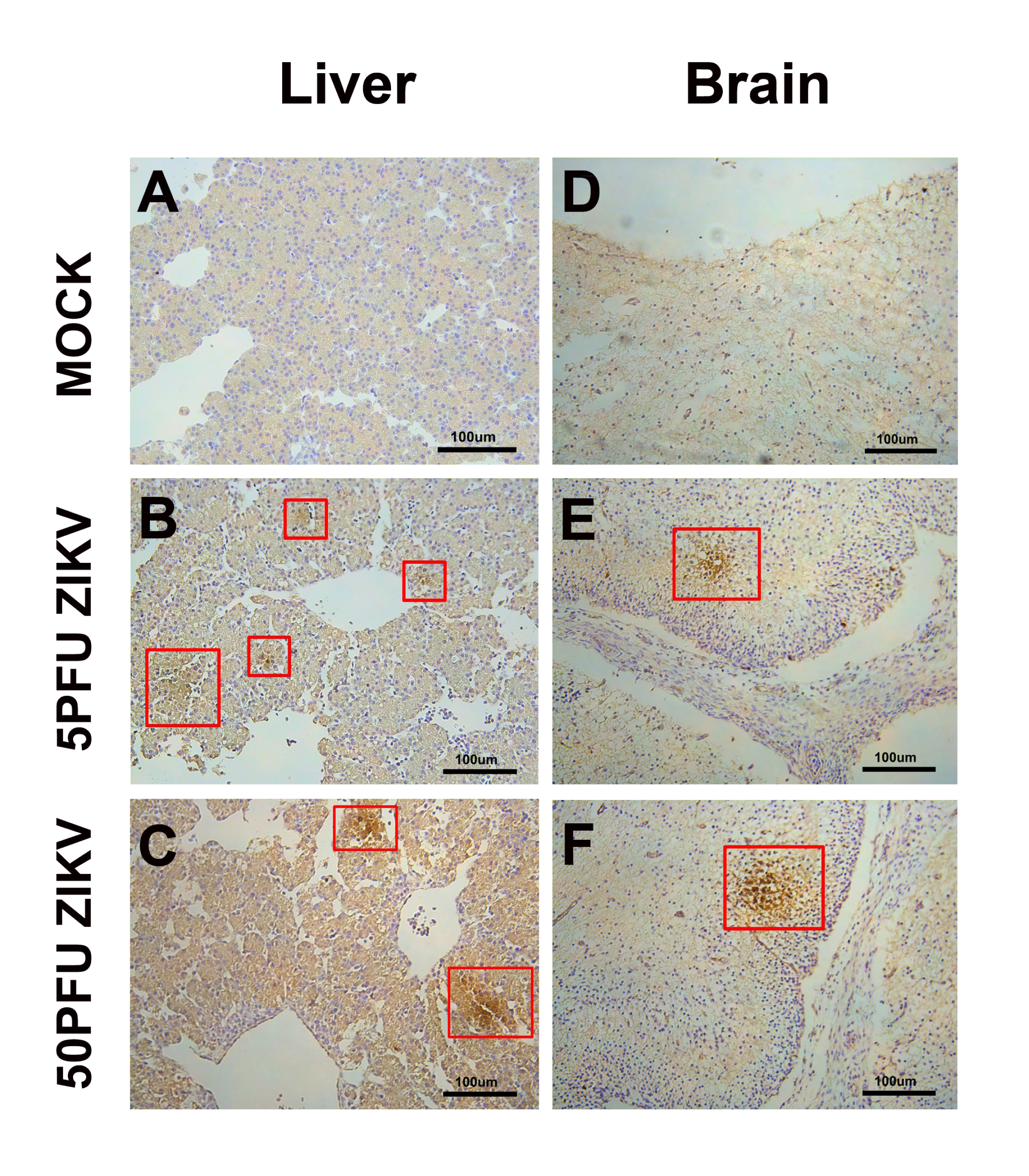

Supplement: FIGURE S2 — ZIKV was detected in chicken embryonic livers with immunohistochemistry. Chicken embryonic livers were harvested at 11 dpi, and immunohistochemical assay was performed to probe ZIKV Envelop protein. In brief, tissue sections at 5 μm were de-waxed in xylene, rehydrated and heated for antigen retrieval. After being immersed in 3% hydrogen peroxide for 25 min to block endogenous peroxidase, the sections were blocked with 3% BSA for 30 min at room temperature and incubated with primary antibodies against ZIKV Envelop protein (1:100, BF-1176-56, BioFront Technologies Inc.,) at 4°C overnight with shaking. The sections were then incubated with HRP labeled secondary antibodies (1:200; Invitrogen) at room temperature for 50 min in a dark box and finally visualized with DAB. All the sections were later counterstained with hematoxylin. A minimum of 3 randomly-selected images from 5 samples were assessed per group. [file Image_2.TIF]

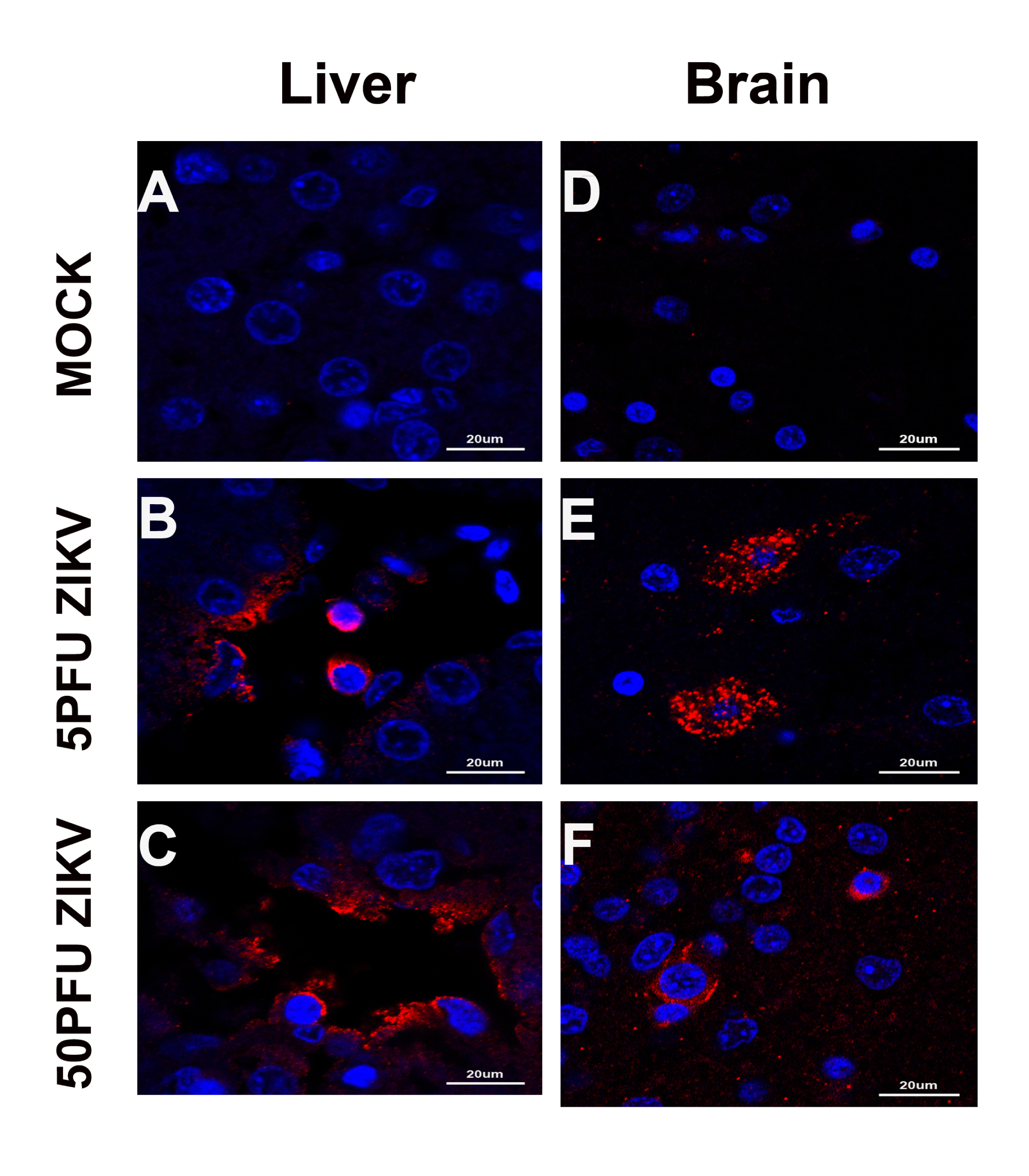

Supplement: FIGURE S3 — ZIKV was detected in chicken embryonic livers with immunefluoresent staining. Chicken embryonic livers were harvested at 11 dpi, and immunofluoresent staining was performed to probe ZIKV Envelop protein. In brief, tissue sections at 5 μm were de-waxed in xylene, rehydrated and heated for antigen retrieval. After being immersed in 3% hydrogen peroxide for 10 min to block endogenous peroxidase, the sections were blocked with 5% inactivated goat serum for 30 min at room temperature and incubated with primary antibodies against ZIKV Envelop protein (1:100, BF-1176-56, BioFront Technologies Inc.,) at 4°C overnight with shaking. The sections were then incubated with the corresponding Alexa Fluor CY3 labelled secondary antibodies (1:300; Invitrogen) at room temperature for 2 hours in a dark box. All the sections were later counterstained with DAPI (1:1000; Invitrogen) at room temperature for 30 min. A minimum of 3 randomly-selected images from 5 samples were assessed per group. [file Image_3.TIF]

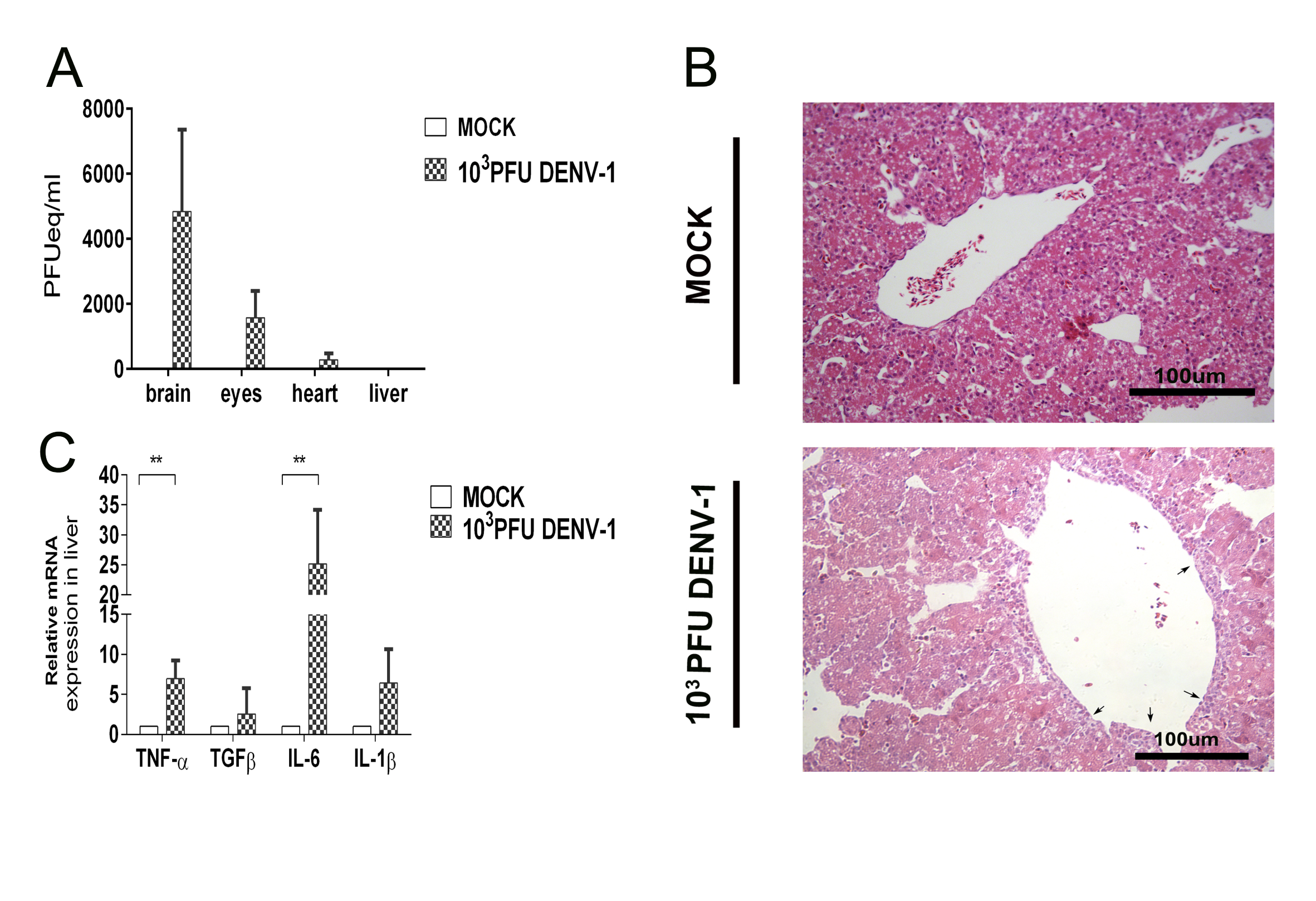

Supplement: FIGURE S4 — DENV-1 replicated in chicken embryos and induced inflammatory response in embryonic livers. Chicken embryos were inoculated with 1000 PFU DENV-1 virus (Hawaii strain, GenBank #: KM204119) with brain injection at E5, and various organs were harvested at 11 dpi for assays. (A) The viral load of DENV-1 in various organs in chicken embryos. Viral RNA was extracted and quantified with qPCR. The viral loads in tissues were calculated by using standard curves obtained from serial dilutions of DENV-1 stocks titrated with plaque assays in Vero cells and expressed as plaque-forming units per milliliter equivalents (PFUeq/ml). (B) Liver tissue was sectioned at 5 μm and HE staining was performed for histological analysis. A large amount of inflammatory cells were observed in perivascular area in DENV-1 infected chicken embryonic liver. (C) Total RNA was extracted from liver tissue and qPCR was performed to determine mRNA expression levels of various inflammation related cytokines. TNF- and IL-6 showed significant increase compared to mock. [file Image_4.TIF]
